# Idiopathic normal pressure hydrocephalus: validation of the DESH score in the Capital Region of Denmark

**DOI:** 10.1007/s00701-026-06913-4

**Published:** 2026-05-13

**Authors:** Bjarni Johannsson, Jan Saip Aunan-Diop, Jonathan Frederik Carlsen, Anika Mai Johannsdottir, Sune Munthe, Frantz Rom Poulsen, Mathias Just Nortvig, Steen Hasselbach, Tina Nørgaard Munch, Christian Bonde Pedersen

**Affiliations:** 1 Department of Neurosurgery, Odense University Hospital, Odense, Fyn 5000 Denmark; 2 Department of Neurosurgery, Copenhagen University Hospital, Copenhagen, Capital Region 2100 Denmark; 3 Department of Congenital Disorders, Statens Serum Institut, Copenhagen, Capital Region 2300 Denmark; 4 Department of Clinical Medicine, University of Copenhagen, Copenhagen, Capital Region 2100 Denmark; 5 Department of Radiology, Copenhagen University Hospital, Copenhagen, Capital Region 2100 Denmark; 6 Department of Neurology, Danish Dementia Research Centre, Copenhagen University Hospital, Copenhagen, Capital Region 2100 Denmark

**Keywords:** Normal pressure hydrocephalus, Adult hydrocephalus, Shunt surgery, Diagnostics

## Abstract

**Objective:**

Previous research has shown that the Disproportionally Enlarged Subarachnoid-space Hydrocephalus (DESH) score may be a prognostic factor for shunt surgery response for idiopathic normal pressure hydrocephalus (iNPH) patients at 1-month follow-up after shunt surgery. The objective of the current study was external validation in an independent cohort with a longer follow-up period. Secondarily, to assess whether the grading system was confounded by imaging modality.

**Methods:**

Preoperative baseline characteristics, magnetic resonance imaging (MRI) and/or computed tomography (CT) scans were retrospectively obtained in 127 shunt-operated iNPH patients. Evans’ index, dilation of Sylvian fissures, tight high convexity, focal sulci, callosal angle, and a combination of these radiologic findings (DESH score) were compared according to patients’ response to shunt treatment at a mean follow-up period of 7.4-months. In addition, the data were adjusted for whether grading was performed by MRI or CT.

**Results:**

Multivariate logistic regression, adjusted for age, sex, and imaging modality, demonstrated a significant association between the DESH score and objective shunt response at follow-up (Odds Ratio 1.35, *p* = 0.036). Bivariate logistic regression revealed no significant difference in DESH score assessments between CT and MRI. Moreover, including or excluding imaging modality in the multivariate model did not meaningfully alter the regression coefficients.

**Conclusion:**

Consistent with and supportive of prior findings, the DESH score is associated with shunt response in iNPH patients. No evidence of confounding by imaging modality was identified in this cohort. However, formal equivalence between CT and MRI for DESH scoring remains to be established in studies with paired imaging.

## Introduction

Idiopathic normal pressure hydrocephalus (iNPH) is a neurological disorder with a currently unknown etiology. The condition is characterized by disrupted cerebrospinal fluid (CSF) dynamics within the brain, resulting in symptomatic ventricular enlargement despite normal intracranial pressure. Individuals with iNPH typically manifest symptoms from the Hakim-Adams triad, which includes gait disturbance, often of the hypokinetic, magnetic type, urinary urgency and/or incontinence, and, predominantly subcortical, cognitive impairment [6]. Since its initial description by Hakim and Adams in 1965 [9], iNPH has posed a significant challenge in modern neurosurgery and neurology, primarily due to the absence of a universally accepted gold standard for diagnosis and therefore treatment. Based on current guidelines, patients are categorized as likely, possibly, or unlikely to have iNPH based on their clinical and radiological findings [19].

Recent studies indicate that the prevalence of iNPH may be higher than previously estimated, particularly among individuals aged 80 years and older, with estimates as high as 8.9% [2, 14]. Given the observed socioeconomic cost–benefit of shunt surgery and improved patient quality of life, understanding and effectively treating iNPH has become increasingly imperative [12, 13, 22].

In the evaluation of patients with iNPH, clinical and radiological assessments serve as the cornerstone, often followed by ancillary tests such as the tap test (TT), lumbar drainage and/or the infusion test (IT), which are commonly employed in European centers. The decision to proceed with surgery varies across different centers, with some relying on clinical and radiological findings only, whereas others integrate ancillary test results into their surgical decision-making. At the Memory Clinic at the Danish Dementia Research Centre, both the TT and IT are routinely utilized to guide decisions regarding surgical candidacy.

Despite the best efforts of clinicians, a critical gap exists in the absence of standardized criteria for shunt selection within this patient population. This is compounded by substantial variability in the predictive values of the TT and IT across existing studies [23].

We aim to expand on previous single-center studies [15, 16] to discern and compare the pre-surgical variables distinguishing shunt responders from non-responders, shedding light on the nuanced factors influencing treatment efficacy in this challenging neurological and neurosurgical condition. The study’s objectives are to validate the predictive value of the Disproportionally Enlarged Subarachnoid-space Hydrocephalus (DESH) [21] score for shunt surgery response in an independent patient cohort from a separate center than those used in the two previous studies, and to assess the potential impact of imaging modality, namely computed tomography (CT) versus magnetic resonance imaging (MRI) on the grading scale.

## Materials and methods

The present cohort is identical to that described in the authors’ previous study, in which the inclusion and exclusion criteria are likewise detailed [10].

All data were recorded from the digital patient files and the PACS system for imaging studies.

Data were retrospectively obtained from the period 2013 to 2020. Inclusion criteria were: 1) diagnosis of probable or possible iNPH according to international guideline criteria [20] 2) a lumbar infusion test as part of the diagnostic evaluation, 3) an available brain CT or MRI scan, performed within one year prior to surgery, and 4) subsequent ventriculoperitoneal shunt implantation, and 5) at least 2 months of clinical follow-up after surgery. If patients fulfilled clinical and neuroimaging criteria for iNPH [20] supplementary diagnostic tests were performed. A lumbar infusion test including a measurement of R_out_ was obtained by an automated CSF infusion test system (CELDA System, Likvor AB, Umeå, Sweden). R_out_ above 16 was regarded as supportive for decisions of shunting. A negative test result was regarded as less conclusive, due to the known low negative predictive power [24]. A negative CSF infusion test was therefore not an exclusion criterion for shunt surgery. Based on these criteria, patients were referred to the Department of Neurosurgery for final evaluation before shunting. In case of an uncertain diagnosis, patients were evaluated at an interdisciplinary conference between clinicians from the Memory Clinic at the Danish Dementia Research Centre and the Department of Neurosurgery at Copenhagen University Hospital before referral to surgery.

Shunt response evaluation was performed at outpatient follow-up visits at the Memory Clinic at the Danish Dementia Research Centre.

Changes in gait and continence scores were evaluated separately using the iNPH scale by Hellström et al. [11] as (1) worsening, increase in score by 1 or more; (2) no effect, no change in scores; (3) moderate effect, a decrease in score by 1; and (4) substantial effect, a decrease in score by 2 or more. Changes in cognition were evaluated as (1) worsening, decrease in MMSE score by 2 or more; (2) no effect, change in score by − 1 to + 1; (3) moderate effect, increase in score by 2; and (4) substantial effect, increase in score by 3 or more. Changes in gait, continence, and MMSE were then scored each on a scale from − 1 to 2: worsening, − 1; no change, 0; moderate effect, 1; and substantial effect, 2; yielding a summary shunt response score from − 3 to 6. Positive response was defined as a summary shunt response score ≥ 1, as outlined by previous research [10].

The primary outcome was summary shunt response score, while the proposed predictors encompassed the DESH score and modality, CT and MRI.

Evans’ index (EI), callosal angle (CA), tight high convexities (THC), focal sulci and dilated Sylvian fissures were quantified by a radiologist, using either CT or MRI, contingent on availability.

EI was defined as the maximum width of the frontal horns of the lateral ventricles divided by the widest skull diameter in the same axial plane [25]. CA was defined as the angle of the lateral ventricles in the paracoronal plane, perpendicular to the anterior–posterior commissure axis [21]. The assessment of THC, focal sulci, and dilated Sylvian fissures was assessed subjectively due to the lack of established objective criteria. These parameters, EI, CA, THC, Sylvian fissure dilation, and the extent of focal sulci, were aggregated into a DESH score [21]. Points ranging from 0 to 2 were assigned based on the extent of each marker, following the criteria delineated by the original authors [21] (Table 1).

**Table 1:** DESH score grading, as depicted in the original paper by Shinoda et al. [21]

Grade	Definition
Evans’ index	
0	≤ 0.3
1	0.31–0.35
2	> 0.35
Callosal angle	
0	≥ 100°
1	90°−99°
2	< 90°
Sylvian fissure	
0	Normal or narrow
1	Slight or unilateral dilation
2	Bilateral dilation
Tight high convexity	
0	Normal or wider than normal
1	Slight compression
2	Definitive compression
Focal sulci dilation	
0	None observed
1	One observed
2	Two or more observed

The radiologist was blinded to the clinical outcome (shunt response) during image evaluation.

## Statistics

Multivariate logistic regression analysis was conducted to assess the defined outcome, shunt response, a dichotomous variable coded as 1 for a positive response. We adjusted for several covariates: age, a continuous variable; sex, a dichotomous variable where 1 represents female; and imaging modality, also dichotomous, with 1 indicating the use of MRI. Given that the study focused on a single hypothesis, unadjusted p-values are reported throughout with α = 0.05.

A second multivariate logistic regression analysis was exploratively conducted without the modality term to assess potential bias in the form of significant altering of coefficients, when introducing the term.

Finally, regression analysis was exploratively performed to assess whether DESH score differed according to imaging modality, to further evaluate the impact of modality.

Descriptive results are presented as frequencies and percentages for binary variables. Continuous and ordinal variables are presented as means and standard deviation (SD).

Regression results are presented as odds ratios (OR), standard errors (SE), 95% confidence intervals (95% CI) and p-values.

All statistical analyses were performed using STATA 16.0 (StataCorp. 2018. Stata Statistical Software: Release 16. College Station, TX: StataCorp LLC).

## Results

In the period, 361 consecutive patients were diagnosed with possible or probable iNPH at the Memory Clinic. Of these, 127 patients met the inclusion criteria. After a mean follow-up period of 7.4 months (range 2–20 months), 82% of patients had a positive response to shunt surgery [10].

Of the 234 patients not included, the principal reasons for non-inclusion were absence of preoperative neuroimaging within the one-year window required, lack of an available lumbar infusion test result, withdrawal of the surgical indication after multidisciplinary review, less than two months of postoperative clinical follow-up at the time of data extraction, or the patient not subsequently undergoing shunt surgery. The diagnosis of possible or probable iNPH was therefore not retracted in the non-included patients; rather, these patients did not meet the predefined criteria for the present analysis.

Descriptive results are shown in Table 2.

**Table 2:** Descriptive analysis

Variable	Description
Age	74.3 ± 6.4
Female sex	46 (36.2%)
MRI	107 (84.3%)
CT	20 (15.7%)
Positive surgery response	104 (81.9%)
Mean DESH score (no response)	5.6 ± 1.7
Mean DESH score (positive response)	6.3 ± 1.7
**DESH score**	
0	0 (0%)
1	0 (0%)
2	3 (2.4%)
3	2 (1.6%)
4	17 (13.4%)
5	22 (17.3%)
6	27 (21.3%)
7	22 (17.3%)
8	26 (20.5%)
9	7 (5.5%)
10	1 (0.8%)
**Tight high convexity**	
None	49 (38.6%)
Slight	41 (32.3%)
Significant	37 (29.1%)
**Dilated sylvian fissure**	
None	18 (14.2%)
Unilateral	44 (34.7%)
Bilateral	65 (51.2%)
**Focal Sulci**	
None	103 (81.1%)
Unilateral	8 (6.3%)
Bilateral	16 (12.6%)
**Corpus callosal angle**	
≥ 100°	8 (6.3%)
90°−99°	10 (7.8%)
< 90°	109 (85.8%)
**Evan’s index**	
≤ 0.3	0 (0.0%)
0.31–0.35	24 (18.9%)
> 0.35	103 (81.1%)

Results are displayed in percentages for categorical variables or mean ± SD (standard deviation) for continuous variables

In a multivariate regression analysis, with adjustment for age, sex, and imaging modality, the DESH score was found to be significantly and positively associated with response to shunt surgery. Each one-unit increase in the DESH score significantly correlated with a 1.35-fold (95% CI 1.02–1.90) higher odds of a positive surgical outcome (Table 3).

**Table 3:** Multiple logistic regression results for shunt effect, including brain imaging modality

Predictor	OR	SE	95% CI	*P*-value
DESH score	1.35	0.20	1.02–1.90	**0.036**
Age	1.0	0.04	0.92–1.07	0.926
Sex	0.49	0.24	0.18–1.28	0.144
Imaging modality	2.19	1.35	0.65–7.36	0.207

*OR* Odds ratio, *SE* standard error, *95% CI* 95% confidence interval

In a second multivariate regression analysis, removing the modality term (CT/MRI), did not significantly alter the coefficient for shunt response, with an odds ratio of 1.35, with modality included, and 1.33 (95% CI 1.02–1.90) excluding modality, a coefficient difference of 1.5% (Table 4).

**Table 4:** Multiple logistic regression results for shunt effect, excluding brain imaging modality

Predictor	OR	SE	95% CI	*P*-value
DESH score	1.33	0.19	1.01–1.76	**0.046**
Age	0.99	0.04	0.92–1.07	0.783
Sex	0.54	0.26	0.21–1.38	0.199

*OR* Odds ratio, *SE* Standard error, *95% CI* 95% confidence interval

In the bivariate regression analysis, modality could not significantly predict the DESH score (Table 5).

**Table 5:** Bivariate logistic regression for brain imaging modality

Predictor	OR	SE	95% CI	*P*-value
DESH score	0.86	0.13	0.64–1.15	0.306

*OR* Odds ratio, *SE* standard error, *95% CI* 95% confidence interval

All 127 patients underwent ventriculoperitoneal shunt implantation; no ventriculoatrial or other shunt configurations were used. Specific valve types and pressure settings were not systematically captured in the present dataset and could therefore not be analysed. Comorbidities and surgical complications for patients overlapping with this cohort have been previously reported in detail in a closely related radiological study from the same center [10], in which major urogenital, musculoskeletal, or neurological comorbidities were registered for 65% of patients with no significant difference between responders and non-responders, and complications within one year were observed in 33% of patients (more frequently in non-responders, 64%, than in responders, 24%; *P* < 0.001). A formal subgroup analysis of the mechanisms of non-response was beyond the scope of the present study.

## Discussion

This study aimed to determine whether the DESH score is significantly associated with shunt surgery response at a second Danish neurosurgical center, namely the Department of Neurosurgery at Copenhagen University Hospital, thereby extending prior validation in patients from the Region of Southern Denmark, and whether the assessment of the DESH score is confounded by imaging modalities (CT and MRI).

Our findings demonstrate a significant correlation between the DESH score and shunt surgery response. Additionally, no evidence of an effect of imaging modality on the DESH–outcome relationship was observed.

iNPH is a partially treatable condition mainly seen in elderly patients. Overlapping symptomatology and neuroimaging findings between iNPH patients and other types of dementia patients, as well as with common age-related conditions, challenge the diagnosis and treatment. For example, gait disturbance may be present in 35% of individuals over the age of 70 years. Combined with the low specificity and the invasiveness of liquor dynamic diagnostic testing (i.e. tap and infusion tests), identifying and predicting potential shunt responders by other means is highly important. Despite recent efforts to standardize the clinical approach, iNPH is often under- and misdiagnosed [18].

The pathophysiology of iNPH is incompletely understood and likely involves altered cerebrospinal fluid dynamics and reduced craniospinal compliance, with isolated hydrodynamic parameters performing inconsistently as predictors of shunt response [4, 5]. Within this pathophysiological context, the DESH score is best understood as an aggregated, image-based marker of the secondary anatomical changes characteristic of the disease, and its clinical value lies in providing a predictor of shunt response that does not depend on resolving the underlying mechanism. Emerging viscoelastic imaging methods, such as MR elastography, may complement DESH by providing further mechanistic insight in future studies [3, 17].

Attempts to identify shunt responders based on DESH show variable results. Some studies suggest that the presence of DESH is correlated to treatment response [7, 21]. Simultaneously, low degree of DESH does not seem to *rule out* shunt response [1, 8]. In the present study, we found a correlation between increased DESH score and treatment response. Thus, we contribute to the increasing load of evidence suggesting that image-based parameters serve as an independent predictor for treatment response. At present, the natural history of DESH is unknown, and longitudinal studies could improve the interpretation of DESH in variable settings.

**Fig. 1:**
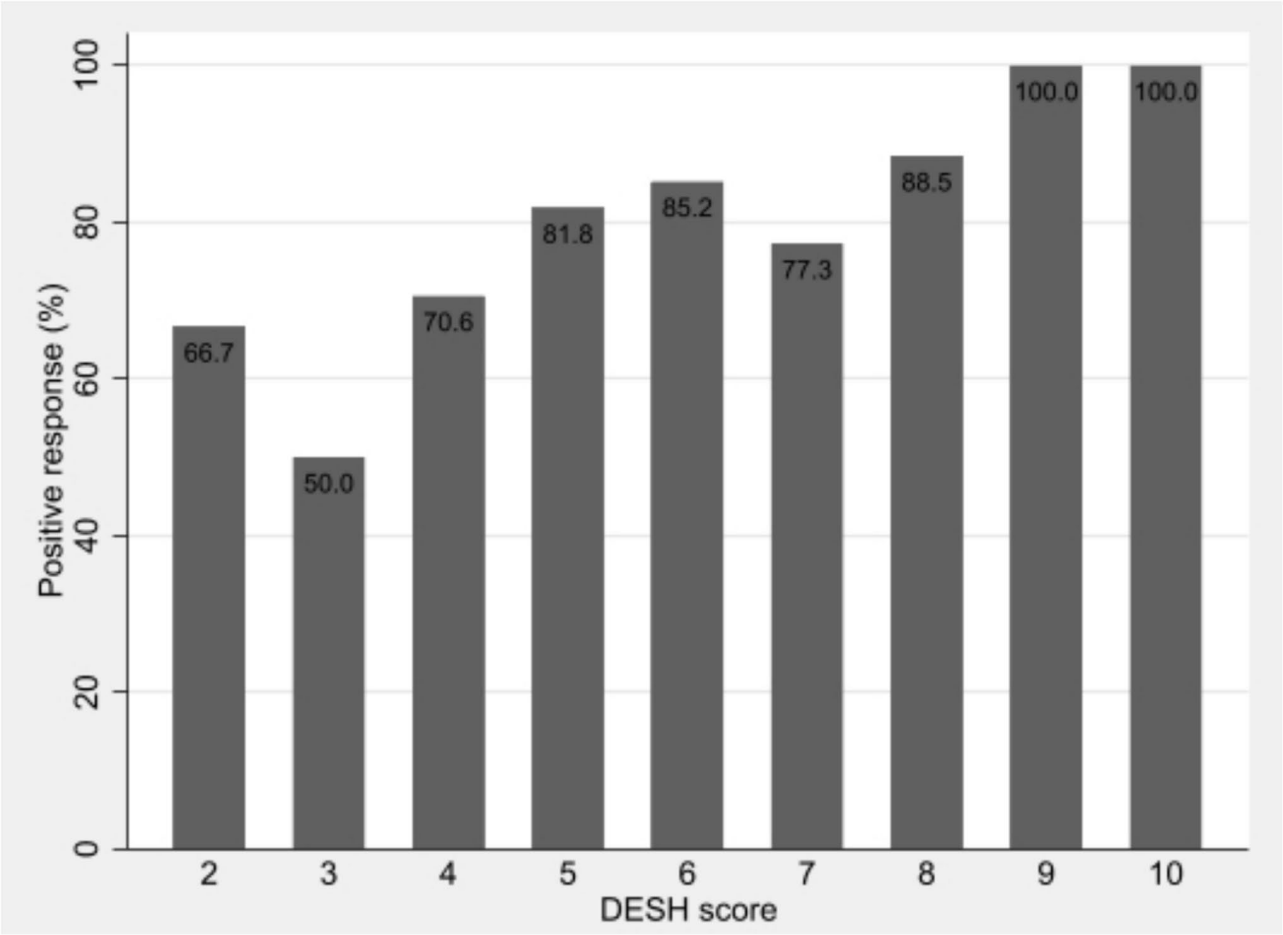
Proportion of patients with a positive shunt response stratified by preoperative DESH score. Note that DESH scores of 0 and 1 were not observed in this cohort. Numbers above bars represent response proportions; absolute patient numbers per group are provided in Table 2

The observed discrepancy in DESH and its correlation with treatment outcomes might be attributable to the natural progression of idiopathic normal pressure hydrocephalus (iNPH). It may be postulated that DESH is a time-dependent finding in iNPH, with an increase over time. As a result, DESH may lack prognostic value in earlier stages of the disease. The observation that shunt-response rate seems to increase with increasing DESH score (Fig. 1) could support this theory. For example, more advanced cases of NPH could be correlated with higher DESH score and more pre-treatment symptoms. Treatment could then have a greater relative effect in relieving those symptoms. Attempts at describing the temporal development of DESH and its relations to symptoms would be of great scientific value. If possible, these studies should be combined with magnetic resonance elastography to assess the correlation between viscoelastic parameters and the development of DESH.

At our center, MRI is routinely preferred as part of the diagnostic workup in harmony with current guidelines [19]. While MRI offers differential diagnostic advantages due to high levels of anatomical detail, MRI may be impractical in certain patients. For example, cardiac pacemakers are a relative contraindication for MRI imaging. Furthermore, MRI is more time consuming and may not be available in peripheral centers. Therefore, newly referred patients may lack a brain MRI. The value of CT versus MRI for performing DESH calculations is therefore an unknown factor relevant in a clinical setting. Our results indicate that the DESH score serves as an independent predictor of outcome, adjusted for imaging modality (Table 4 and 5). CT may serve as a reliable alternative to MRI for the initial evaluation of suspected iNPH, but MRI still offers important differential diagnostic information, and should be done before shunting patients, if possible.

## Limitations and strengths

A key limitation is the preselection of patients based on prior tap test or infusion testing, resulting in a cohort with a high pretest probability of treatment response and introducing workup (selection) bias. This enriches the cohort for individuals already suspected of CSF dynamic disturbances, thereby limiting generalizability and potentially inflating the observed association between DESH score and clinical outcomes.

A direct comparison between CT and MRI would require within-patient paired imaging, which was not available in this study. Therefore, the lack of association between imaging modality and the DESH–outcome relationship suggests no evidence of confounding by modality in this cohort, but does not imply equivalence between CT and MRI. A dedicated within-patient comparison of CT- and MRI-based DESH scoring in a contemporary subset of patients with both modalities available is therefore an essential next step before CT can be recommended as a fully equivalent alternative to MRI for DESH grading. We further note that the DESH score was scored only in the 127 included patients and not in the full screened population of 361 patients; the differential diagnostic value of DESH for distinguishing iNPH from other diagnoses considered at the Memory Clinic could therefore not be evaluated in the present dataset and should be addressed in dedicated prospective studies including non-shunted comparator groups.

Diagnostic misclassification remains an inherent challenge in iNPH research, as clinical and radiological features overlap with other neurodegenerative and age-related conditions such as Alzheimer’s disease and Parkinson’s disease. Consequently, some patients in the cohort may have been misclassified, potentially attenuating observed associations. Additionally, radiological features included in the DESH score may be present across a spectrum of conditions, which should be considered when interpreting its prognostic value.

Strengths include the relatively large, well-characterized cohort, blinded radiological assessment, and external validation of prior findings in an independent clinical setting. Additionally, the analysis demonstrates robustness of the DESH score across imaging modalities, supporting its applicability in routine clinical practice.

## Conclusion

Consistent with and supportive of prior findings, the DESH score is associated with shunt response in iNPH patients. No evidence of confounding by imaging modality was identified in this cohort. However, formal equivalence between CT and MRI for DESH scoring remains to be established in studies with paired imaging.

## Data Availability

No datasets were generated or analysed during the current study.
